# Supplementary material for: Chimpanzees make tactical use of high elevation in territorial contexts
Source: PLoS Biol. 2023 Nov 2;21(11):e3002350. doi: 10.1371/journal.pbio.3002350 (PMC10621857; doi:10.1371/journal.pbio.3002350)
Supplement: S1 Table — Results of the full model including the interaction between one’s own adult party size and intercommunity distance. (DOCX) [file pbio.3002350.s001.docx]

**S1 Table**. **Determinants of stopping events at peripheral hills.**

Results of the *full model* including the interaction between one’s own adult party size and inter-community distance.

| **Terms** | **Estimate (SE)** | **z value** | **P value** | **95% CI** | **Mean (SD)** |
| --- | --- | --- | --- | --- | --- |
| (Intercept) | -1.104 (0.292) | -3.772 | (i) | -1.724; -0.579 | NA |
| Movement toward border ^b, d^ | 1.709 (0.286) | 5.957 | **< 0.001** | 1.283; 2.364 | NA |
| Nb. Hills used before ^a, b^ | -0.104 (0.139) | -0.754 | 0.450 | -0.403; 0.100 | 1.34 (1.85) |
| Nb. Hills used after ^a, b^ | -0.417 (0.119) | -3.499 | **< 0.001** | -0.658; -0.256 | 1.35 (1.91) |
| Own party size ^a, b^ | 0.092 (0.103) | 0.895 | (i) | -0.086; 0.298 | 6.12 (4.33) |
| Inter-community distance ^a, b^ | -0.020 (0.108) | -0.185 | (i) | -0.214; 0.201 | 4447 (1647) |
| Own party size * Inter-community distance ^b^ | 0.084 (0.108) | 0.783 | 0.433 | -0.085; 0.264 | NA |
| Relative distance to center ^a, c^ | 0.104 (0.129) | 0.807 | 0.419 | -0.160; 0.377 | 0.085 (0.02) (0.020) |
| Quadratic relative distance to center ^c^ | 0.016 (0.063) | 0.266 | 0.789 | -0.118; 0.145 | NA |
| Elevation ^a, c^ | -0.214 (0.122) | -1.745 | 0.081 | -0.482; -0.006 | 238 (9) |
| Location ^a c, e^ | 0.232 (0.129) | 1.793 | 0.073 | -0.027; 0.479 | 62 (21) |
| Time of the day ^a, c, f^ | -0.293 (0.106) | -2.743 | **0.006** | -0.478; -0.099 | 12.66 (3.12) |
| Sex (Males as reference) ^c, g^ | -0.222 (0.199) | -1.114 | 0.265 | -0.621; 0.164 | NA |
| Group (South) ^c, h^ | -0.391 (0.290) | -1.350 | 0.176 | -0.994; 0.214 | NA |
| Temporal autocorrelation term ^a, c^ | -0.077 (0.107) | -0.715 | 0.474 | -0.249; 0.133 | NA |

(a) z-transformed; (b) test predictors; (c) control predictors; (d) toward border as compared to toward center; (e) location refers to kernel values extracted from utilization distribution based on the track logs; kernel values increase with the distance to the territory center;(f) circadian values; (g) refers to males as compared to females; (h) refers to South group as compared to East group; (i) have no meaningful interpretation. Data set n = 717; two groups (East and South); Marginal effect sizes (R²):0.132; conditional R2: 0.507. P-values in **bold** indicate a statistically significant effect (α = 0.05). Dispersion parameter = 0.74, χ ² =524.12, df =702, P = 0.99. Largest VIF = 1.34.
