# Supplementary material for: Chimpanzees make tactical use of high elevation in territorial contexts
Source: PLoS Biol. 2023 Nov 2;21(11):e3002350. doi: 10.1371/journal.pbio.3002350 (PMC10621857; doi:10.1371/journal.pbio.3002350)
Supplement: S2 Table — Results of a reduced model, lacking statistically nonsignificant interactions. (DOCX) [file pbio.3002350.s002.docx]

**S2 Table**. **Determinants of stopping events at peripheral hills.**

Results of a *reduced model*, lacking statistically non-significant interactions.

| **Terms** | **Estimate (SE)** | **z value** | **P value** | **95% CI** |
| --- | --- | --- | --- | --- |
| (Intercept) | -1.125 (0.287) | -3.915 | (i) | -1.749; -0.715 |
| Movement toward border ^b, d^ | 1.656 (0.283) | 5.848 | **< 0.001** | 1.246; 2.198 |
| Nb. Hills used before ^a, b^ | -0.071 (0.134) | -0.531 | 0.595 | -0.387; 0.179 |
| Nb. Hills used after ^a, b^ | -0.381 (0.114) | -3.338 | **< 0.001** | -0.590; -0.185 |
| Own party size ^a, b^ | 0.079 (0.100) | 0.785 | 0.432 | -0.088; 0.265 |
| Inter-community distance ^a, b^ | -0.023 (0.106) | -0.225 | 0.822 | -0.188; 0.164 |
| Relative distance to center ^a, c^ | 0.147 (0.124) | 1.186 | 0.235 | -0.083; 0.402 |
| Quadratic relative distance to center ^c^ | 0.017 (0.060) | 0.284 | 0.776 | -0.112; 0.167 |
| Elevation ^a, c^ | -0.206 (0.118) | -1.739 | 0.081 | -0.448; 0.023 |
| Location ^a, c, e^ | 0.201 (0.124) | 1.621 | 0.105 | -0.033; 0.390 |
| Time of the day ^a, c, f^ | -0.302 (0.105) | -2.878 | **0.004** | -0.522; -0.145 |
| Sex (Males as reference) ^c, g^ | -0.233 (0.197) | -1.183 | 0.236 | -0.666; 0.125 |
| Group (South) ^c, h^ | -0.269 (0.269) | -0.998 | 0.318 | -0.813; 0.302 |
| Temporal autocorrelation term ^a, c^ | 0.156 (0.095) | 1.641 | 0.100 | 0.034; 0.338 |

(a) z-transformed; (b) test predictors; (c) control predictors; (d) toward border as compared to toward center; (e) location refers to kernel values extracted from utilization distribution based on the track logs; kernel values increase with the distance to the territory center; (f) circadian values; (g) refers to males as compared to females; (h) refers to South group as compared to East group; (i) have no meaningful interpretation. Data set n = 717; two groups (East and South); Marginal effect sizes (R²): 0.134; conditional R2: 0.489. P-values in **bold** indicate a statistically significant effect (α = 0.05). Dispersion parameter = 0.77, χ ² = 542.93, df = 703, P = 0.99.
