# Supplementary material for: Chimpanzees make tactical use of high elevation in territorial contexts
Source: PLoS Biol. 2023 Nov 2;21(11):e3002350. doi: 10.1371/journal.pbio.3002350 (PMC10621857; doi:10.1371/journal.pbio.3002350)
Supplement: S3 Table — Percentages are calculated within each territorial section. (DOCX) [file pbio.3002350.s003.docx]

**S3 Table.** **Activity budgets (percentage of observation time) across four sections of the territories (core area, kernel < 25; post-core area, kernel 25 to 50; pre-periphery, kernel 50 to 75; periphery, kernel > 75) for both groups and per group. Percentages are calculated within each territorial section.**

|  |  | **Core area** | **Post-core area** | **Pre-periphery** | **Periphery** |
| --- | --- | --- | --- | --- | --- |
| **Both groups** | **Resting** | 36.84 | 39.92 | 41.43 | 40.67 |
|  | **Feeding** | 45.49 | 40.74 | 37.93 | 35.83 |
|  | **Traveling** | 17.67 | 19.34 | 20.64 | 23.5 |
| **South group** | **Resting** | 40.52 | 41.64 | 43.99 | 42.31 |
|  | **Feeding** | 41.88 | 39.37 | 34.91 | 34.79 |
|  | **Traveling** | 17.6 | 18.99 | 21.1 | 22.9 |
| **East group** | **Resting** | 32.98 | 37.82 | 38.79 | 38.83 |
|  | **Feeding** | 49.28 | 42.42 | 41.06 | 37.01 |
|  | **Traveling** | 17.74 | 19.76 | 20.15 | 24.16 |
