# Supplementary material for: Chimpanzees make tactical use of high elevation in territorial contexts
Source: PLoS Biol. 2023 Nov 2;21(11):e3002350. doi: 10.1371/journal.pbio.3002350 (PMC10621857; doi:10.1371/journal.pbio.3002350)
Supplement: S4 Table — Results of the full model including the interaction between location and elevation. (DOCX) [file pbio.3002350.s004.docx]

**S4 Table**. **The effect of the location and elevation within the whole territory on chimpanzee *resting* activity.** Results of the *full model* including the interaction between location and elevation.

| **Terms** | **Estimate (SE)** | **z-value** | **P value** | **95% CI** | **Mean (SD)** |
| --- | --- | --- | --- | --- | --- |
| (Intercept) | -0.722 (0.034) | -20.879 | (h) | -0.777; -0.651 | NA |
| Location ^a, b, d^ | 0.071 (0.021) | 3.374 | (h) | 0.029; 0.109 | 51 (25) |
| Elevation ^a, b^ | 0.063 (0.022) | 2.800 | (h) | 0.015; 0.102 | 193 (13) |
| Location*Elevation ^b^ | 0.045 (0.018) | 2.457 | **0.014** | 0.014; 0.072 | NA |
| Party size ^a, c^ | 0.172 (0.024) | 7.080 | **< 0.001** | 0.126; 0.225 | 7.35 (5.51) |
| Number of swelling females ^a, c^ | -0.046 (0.018) | -2.445 | **0.014** | -0.084; -0.015 | 0.87 (1.22) |
| Food availability ^a, c^ | -0.014 (0.024) | -0.578 | 0.563 | -0.061; 0.023 | 1.54 (1) |
| Sex of the focal individual_males ^c, e^ | 0.144 (0.036) | 3.980 | **< 0.001** | 0.057; 0.205 | NA |
| Sex of the focal individual_oestrus ^c, f^ | 0.015 (0.145) | 0.109 | 0.913 | -0.205; 0.267 | NA |
| Sin(date) ^c^ | 0.237 (0.027) | 8.696 | (h) | 0.186; 0.286 | NA |
| Cos(date) ^c^ | -0.141 (0.031) | -4.428 | (h) | -0.200; -0.068 | NA |
| Group_South ^c, g^ | 0.241 (0.037) | 6.475 | **< 0.001** | 0.167; 0.316 | NA |

(a) z-transformed; (b) test predictors; (c) control predictors; (d) location refers to kernel values extracted from utilization distribution based on the track logs; kernel values increase with the distance to the territory center; (e) refers to males as compared to females; (f) refers to focal females in oestrus as compared to females; (g) refers to South group as compared to East group; (h) have no meaningful interpretation. Data set n = 42,385 minute-points; two groups (East and South); Marginal effect sizes (R²): 0.017; conditional R2: 0.261. P-values in **bold** indicate a statistically significant effect (α = 0.05). Dispersion parameter = 1.00, χ ² = 91622, df = 91551, P = 0.43. Largest VIF = 1.07.
