# Supplementary material for: Chimpanzees make tactical use of high elevation in territorial contexts
Source: PLoS Biol. 2023 Nov 2;21(11):e3002350. doi: 10.1371/journal.pbio.3002350 (PMC10621857; doi:10.1371/journal.pbio.3002350)
Supplement: S5 Table — Results of the full model including the interaction between location and elevation. (DOCX) [file pbio.3002350.s005.docx]

**S5 Table**. **The effect of the territorial location and elevation on chimpanzee *feeding* activity.**

Results of the *full model* including the interaction between location and elevation.

| **Terms** | **Estimate (SE)** | **z-value** | **P value** | **95% CI** |
| --- | --- | --- | --- | --- |
| (Intercept) | -0.227 (0.033) | -6.847 | (h) | -0.286; 0.169 |
| Location ^a, b, d^ | -0.156 (0.017) | -8.842 | (h) | -0.188; -0.117 |
| Elevation ^a, b^ | 0.037 (0.019) | 1.917 | (h) | 0.002; 0.078 |
| Location*Elevation ^b^ | -0.017 (0.020) | -0.864 | 0.387 | -0.055; 0.026 |
| Party size ^a, c^ | -0.037 (0.018) | -2.034 | **0.041** | -0.064; -0.005 |
| Number of swelling females ^a, c^ | -0.058 (0.018) | -3.153 | **0.001** | -0.091; -0.026 |
| Food availability ^a, c^ | -0.013 (0.023) | -0.580 | 0.562 | -0.052; 0.036 |
| Sex of the focal individual_males ^c, e^ | -0.259 (0.035) | -7.284 | **< 0.001** | -0.311; -0.190 |
| Sex of the focal individual_oestrus ^c, f^ | -0.181 (0.143) | -1.268 | 0.204 | -0.479; 0.079 |
| Sin(date) ^c^ | -0.225 (0.026) | -8.447 | (h) | -0.280; -0.172 |
| Cos(date) ^c^ | -0.082 (0.031) | -2.657 | (h) | -0.133; -0.028 |
| Group_South ^c, g^ | -0.233 (0.036) | -6.388 | **< 0.001** | -0.302; -0.169 |

(a) z-transformed; (b) test predictors; (c) control predictors; (d) location refers to kernel values extracted from utilization distribution based on the track logs; kernel values increase with the distance to the territory center; (e) refers to males as compared to females; (f) refers to focal females in oestrus as compared to females; (g) refers to South group as compared to East group; (h) have no meaningful interpretation. Data set n = 42,385 minute-points; two groups (East and South); Marginal effect sizes (R²): 0.020; conditional R2: 0.192. P-values in **bold** indicate a statistically significant effect (α = 0.05). Dispersion parameter = 0.99, χ ² = 91097, df = 91551, P = 0.85. Largest VIF = 1.07.
