# Supplementary material for: Chimpanzees make tactical use of high elevation in territorial contexts
Source: PLoS Biol. 2023 Nov 2;21(11):e3002350. doi: 10.1371/journal.pbio.3002350 (PMC10621857; doi:10.1371/journal.pbio.3002350)
Supplement: S6 Table — Results of the reduced model lacking the nonsignificant interaction. (DOCX) [file pbio.3002350.s006.docx]

**S6 Table**. **The effect of the territorial location and elevation on chimpanzee *feeding* activity.**

Results of the *reduced model* lacking the non-significant interaction.

| **Terms** | **Estimate (SE)** | **z-value** | **P value** | **95% CI** |
| --- | --- | --- | --- | --- |
| (Intercept) | -0.226 (0.032) | -6.864 | (h) | -0.294; -0.140 |
| Location ^a, b, d^ | -0.155 (0.017) | -8.918 | **< 0.001** | -0.189; -0.120 |
| Elevation ^a, b^ | 0.030 (0.018) | 1.646 | 0.099 | 0.0005; 0.065 |
| Party size ^a, c^ | -0.037 (0.018) | -2.052 | **0.040** | -0.068; -0.001 |
| Number of swelling females ^a, c^ | -0.059 (0.018) | -3.229 | **0.001** | -0.092; -0.017 |
| Food availability ^a, c^ | -0.011 (0.023) | -0.497 | 0.619 | -0.054; 0.031 |
| Sex of the focal individual_males ^c, e^ | -0.253 (0.035) | -7.183 | **< 0.001** | -0.318; -0.192 |
| Sex of the focal individual_oestrus ^c, f^ | -0.176 (0.142) | -1.237 | 0.216 | -0.433; 0.087 |
| Sin(date) ^c^ | -0.222 (0.026) | -8.402 | (h) | -0.275; -0.169 |
| Cos(date) ^c^ | -0.083 (0.030) | -2.695 | (h) | -0.138; -0.022 |
| Group_South ^c, g^ | -0.229 (0.036) | -6.338 | **< 0.001** | -0.325; -0.161 |

(a) z-transformed; (b) test predictors; (c) control predictors; (d) location refers to kernel values extracted from utilization distribution based on the track logs; kernel values increase with the distance to the territory center; (e) refers to males as compared to females; (f) refers to focal females in oestrus as compared to females; (g) refers to South group as compared to East group; (h) have no meaningful interpretation. Data set n = 42,385 minute-points; two groups (East and South); Marginal effect sizes (R²): 0.018; conditional R2: 0.247. P-values in **bold** indicate a statistically significant effect (α = 0.05). Dispersion parameter = 0.99, χ ² = 91097, df = 91551, P = 0.85.
