# Supplementary material for: Chimpanzees make tactical use of high elevation in territorial contexts
Source: PLoS Biol. 2023 Nov 2;21(11):e3002350. doi: 10.1371/journal.pbio.3002350 (PMC10621857; doi:10.1371/journal.pbio.3002350)
Supplement: S7 Table — Results of the full model including the interaction between location and elevation. (DOCX) [file pbio.3002350.s007.docx]

**S7 Table**. **The effect of the territorial location and elevation on chimpanzee *traveling* activity.**

Results of the *full model* including the interaction between location and elevation.

| **Terms** | **Estimate (SE)** | **z-value** | **P value** | **95% CI** |
| --- | --- | --- | --- | --- |
| (Intercept) | -1.613 (0.040) | -40.167 | (h) | -1.680; -1.521 |
| Location ^a, b, d^ | 0.111 (0.021) | 5.173 | (h) | 0.058; 0.159 |
| Elevation ^a, b^ | -0.184 (0.033) | -5.450 | (h) | -0.239; -0.118 |
| Location*Elevation ^b^ | 0.014 (0.023) | 0.629 | 0.529 | -0.024; 0.066 |
| Party size ^a, c^ | -0.249 (0.028) | -8.844 | **< 0.001** | -0.307; -0.186 |
| Number of swelling females ^a, c^ | 0.138 (0.022) | 6.193 | **< 0.001** | 0.100; 0.183 |
| Food availability ^a, c^ | 0.026 (0.026) | 1.018 | 0.309 | -0.026; 0.070 |
| Sex of the focal individual_males ^c, e^ | 0.176 (0.042) | 4.185 | **< 0.001** | 0.112; 0.256 |
| Sex of the focal individual_oestrus ^c, f^ | 0.242 (0.164) | 1.473 | 0.141 | -0.063; 0.513 |
| Sin(date) ^c^ | -0.013 (0.030) | -0.450 | (h) | -0.067; 0.057 |
| Cos(date) ^c^ | 0.332 (0.036) | 9.075 | (h) | 0.249; 0.409 |
| Group_South ^c, g^ | -0.022 (0.042) | -0.513 | 0.608 | -0.104; 0.059 |

(a) z-transformed; (b) test predictors; (c) control predictors; (d) location refers to kernel values extracted from utilization distribution based on the track logs; kernel values increase with the distance to the territory center; (e) refers to males as compared to females; (f) refers to focal females in oestrus as compared to females; (g) refers to South group as compared to East group; (h) have no meaningful interpretation. Data set n = 42,385 minute-points; two groups (East and South); Marginal effect sizes (R²): 0.023; conditional R2: 0.229. P-values in **bold** indicate a statistically significant effect (α = 0.05). Dispersion parameter = 0.98, χ ² = 90576, df = 91551, P = 0.98. Largest VIF = 1.07.
