# Supplementary material for: Chimpanzees make tactical use of high elevation in territorial contexts
Source: PLoS Biol. 2023 Nov 2;21(11):e3002350. doi: 10.1371/journal.pbio.3002350 (PMC10621857; doi:10.1371/journal.pbio.3002350)
Supplement: S8 Table — Results of the reduced model lacking the nonsignificant interaction. (DOCX) [file pbio.3002350.s008.docx]

**S8 Table**. **The effect of the territorial location and elevation on chimpanzee *traveling* activity.**

Results of the *reduced model* lacking the non-significant interaction.

| **Terms** | **Estimate (SE)** | **z-value** | **P value** | **95% CI** |
| --- | --- | --- | --- | --- |
| (Intercept) | -1.605 (0.039) | -40.304 | (h) | -1.685; -1.517 |
| Location ^a, b, d^ | 0.111 (0.021) | 5.286 | **< 0.001** | 0.070; 0.147 |
| Elevation ^a, b^ | -0.188 (0.033) | -5.629 | **< 0.001** | -0.249; -0.122 |
| Party size ^a, c^ | -0.246 (0.027) | -8.837 | **< 0.001** | -0.299; -0.188 |
| Number of swelling females ^a, c^ | 0.140 (0.022) | 6.235 | **< 0.001** | 0.094; 0.173 |
| Food availability ^a, c^ | 0.027 (0.026) | 1.046 | 0.295 | -0.018; 0.072 |
| Sex of the focal individual_males ^c, e^ | 0.174 (0.041) | 4.172 | **< 0.001** | 0.073; 0.257 |
| Sex of the focal individual_oestrus ^c, f^ | 0.236 (0.163) | 1.445 | 0.149 | -0.096; 0.532 |
| Sin(date) ^c^ | -0.013 (0.030) | -0.452 | (h) | -0.061; 0.048 |
| Cos(date) ^c^ | 0.333 (0.036) | 9.161 | (h) | 0.278; 0.398 |
| Group_South ^c, g^ | -0.021 (0.042) | -0.499 | 0.618 | -0.097; 0.061 |

(a) z-transformed; (b) test predictors; (c) control predictors; (d) location refers to kernel values extracted from utilization distribution based on the track logs; kernel values increase with the distance to the territory center; (e) refers to males as compared to females; (f) refers to focal females in oestrus as compared to females; (g) refers to South group as compared to East group; (h) have no meaningful interpretation. Data set n = 42,385 minute-points; two groups (East and South); Marginal effect sizes (R²): 0.024; conditional R2: 0.218. P-values in **bold** indicate a statistically significant effect (α = 0.05). Dispersion parameter = 0.98, χ ² = 90576 df = 91551, P = 0.98.
