# Supplementary material for: Chimpanzees make tactical use of high elevation in territorial contexts
Source: PLoS Biol. 2023 Nov 2;21(11):e3002350. doi: 10.1371/journal.pbio.3002350 (PMC10621857; doi:10.1371/journal.pbio.3002350)
Supplement: S9 Table — Results of the full model including the interactions. (DOCX) [file pbio.3002350.s009.docx]

**S9 Table.** **Determinants of the probability to advance towards rivals’ location after departure from the sampled locations (low and high elevation).**

Results of the *full model* including the interactions*.*

| **Terms** | **Estimate (SE)** | **z value** | **P value** | **95% CI** | **Mean (SD** |
| --- | --- | --- | --- | --- | --- |
| (Intercept) | -0.302 (0.193) | -1.561 | (i) | -0.682; 0.077 | NA |
| Nb. Hills used before ^a, b^ | -0.128 (0.106) | -1.211 | 0.226 | -0.336; 0.079 | 0.76 (1.30) |
| Nb. Hills used after ^a, b^ | -0.016 (0.100) | -0.160 | 0.872 | -0.213; 0.181 | 0.89 (1.44) |
| Own movement ^a, b^ | 0.748 (0.104) | 7.166 | **<0.001** | 0.543; 0.952 | 0.66 (0.47) |
| Imbalance of power ^a, b^ | 0.260 (0.094) | 2.767 | (i) | 0.075; 0.444 | 0.86 (5.50) |
| Inter-community distance ^a, b, d^ | 0.029 (0.102) | 0.288 | (i) | -0.171; 0.230 | 8.29 (0.44) |
| Elevation ^a, b^ | -0.184 (0.104) | -1.776 | (i) | -0.388; 0.019 | 205 (31) |
| Imbalance of power * Inter-community distance ^b^ | -0.008 (0.097) | -0.087 | (i) | -0.200; 0.183 | NA |
| Imbalance of power * Elevation ^b^ | 0.018 (0.093) | 0.201 | (i) | -0.165; 0.202 | NA |
| Inter-community distance * Elevation ^b^ | 0.194 (0.099) | 1.950 | (i) | -0.001; 0.389 | NA |
| Imbalance of power * Inter-community distance * Elevation ^b^ | 0.025 (0.094) | 0.268 | 0.788 | -0.160; 0.210 | NA |
| Relative distance to center ^a, c^ | -0.176 (0.118) | -1.480 | 0.138 | -0.409; 0.057 | 0.08 (0.02) |
| Quadratic relative distance to center ^c^ | 0.074 (0.048) | 1.541 | 0.123 | -0.020; 0.168 | NA |
| Location ^a, c, e^ | -0.079 (0.100) | -0.786 | 0.432 | -0.276; 0.118 | 65 (20) |
| Time of the day ^a, c, f^ | -0.028 (0.095) | -0.296 | 0.767 | -0.216; 0.159 | 11.86 (3.06) |
| Sex (Males as reference) ^c, g^ | -0.040 (0.185) | -0.219 | 0.827 | -0.404; 0.323 | NA |
| Group (South) ^c, h^ | -0.031 (0.207) | -0.152 | 0.878 | -0.438; 0.375 | NA |
| Temporal autocorrelation term ^a, c^ | 0.207 (0.096) | 2.142 | **0.032** | 0.017; 0.396 | (h) |

(a) z-transformed; (b) test predictors; (c) control predictors; (d) natural logarithmic transformed; (e) measured as kernel values from utilization distributions based on the track logs; kernel values increase with the distance to the territory center; (f) circadian values; (g) refers to males as compared to females; (h) refers to South group as compared to East group; (i) have no meaningful interpretation. Data set N = 625, two groups (East and South), marginal effect sizes (R²): 0.184 and conditional R2: 0.209. P-values in **bold** a statistically significant effect (α = 0.05). Largest VIF = 1.76.
