# Supplementary material for: Chimpanzees make tactical use of high elevation in territorial contexts
Source: PLoS Biol. 2023 Nov 2;21(11):e3002350. doi: 10.1371/journal.pbio.3002350 (PMC10621857; doi:10.1371/journal.pbio.3002350)

**S1 Fig.** **Spatial distribution of the low elevation locations (< 180 m asl) within the reconstructed topography** **(meters above sea level) of the territories of South group (left) and East group (right).** The red line represents 95% of the kernel distribution for South group, the blue line the 95% of the kernel distribution for East group. The black lines depict the peripheral area, with lower locations below 180m used by South group in dark red and used by East group in blue.


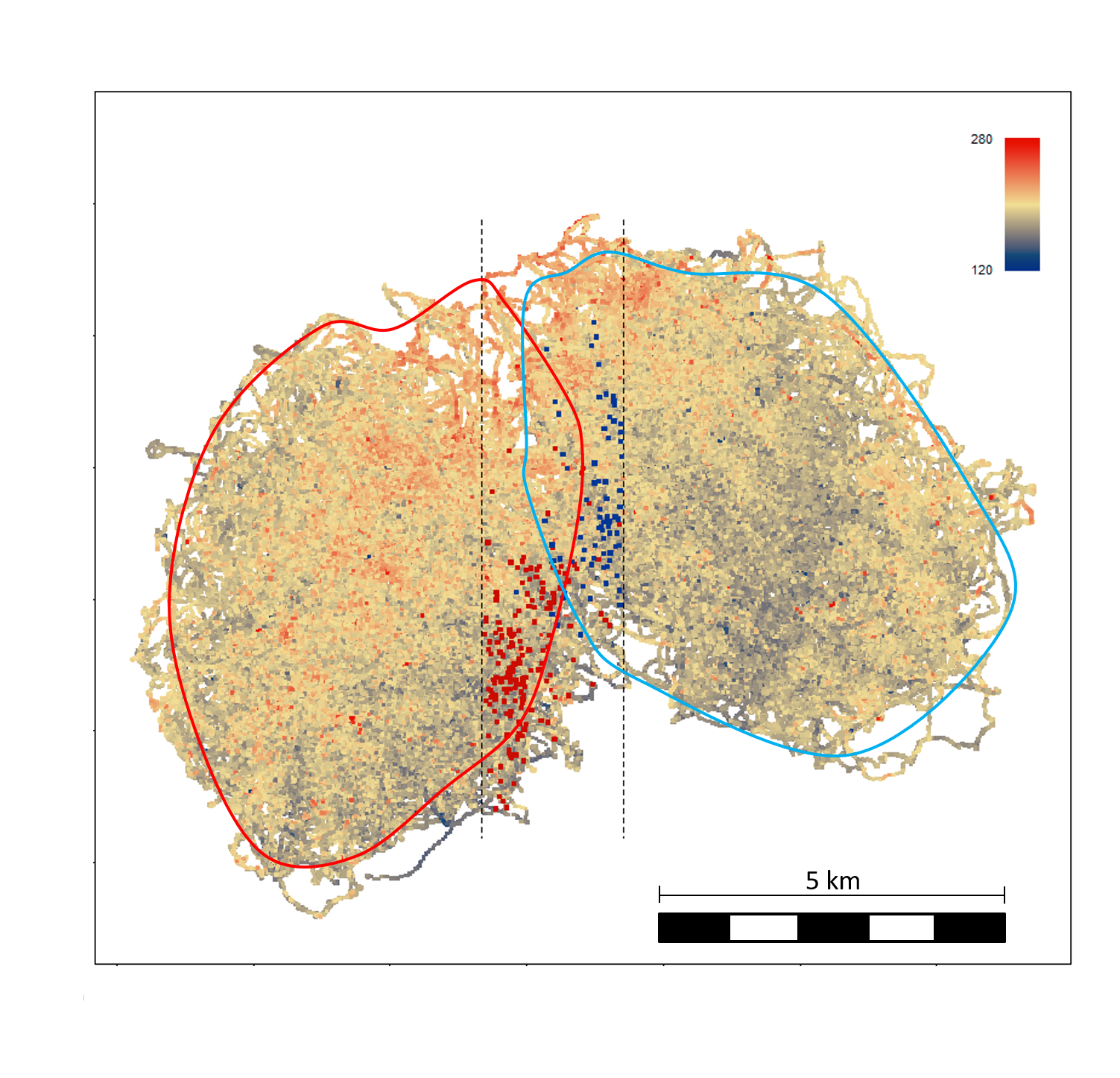

Supplement: S1 Fig — The red line represents 95% of the kernel distribution for South group, the blue line the 95% of the kernel distribution for East group. The black lines depict the peripheral area, with lower locations below 180 m used by South group in dark red and used by East group in blue. (DOCX) [file pbio.3002350.s010.docx]
