# Supplementary material for: Chimpanzees make tactical use of high elevation in territorial contexts
Source: PLoS Biol. 2023 Nov 2;21(11):e3002350. doi: 10.1371/journal.pbio.3002350 (PMC10621857; doi:10.1371/journal.pbio.3002350)

**S2 Fig.** **Distribution of average (red), minimum (green) and maximum (blue) elevation (m asl: meters above sea level) as function of the location in the territory, for South (A) and East (B) groups**. Kernel values on the left of the x-axis correspond to locations near the territory center, while larger kernel values correspond to locations near the border. Each dot corresponds to the average, maximum or minimum value per kernel across all data points in all days, the line depicts the locally weighted smoothing regression line between kernel and elevation, while the ribbon corresponds to the 95% confidence interval. Plots are constructed using a smoothing interpolation of the relationship between kernels and elevation with locally weighted scatterplot smoothing (Lowess). The raw data underlying this Figure may be found in S2 Data.


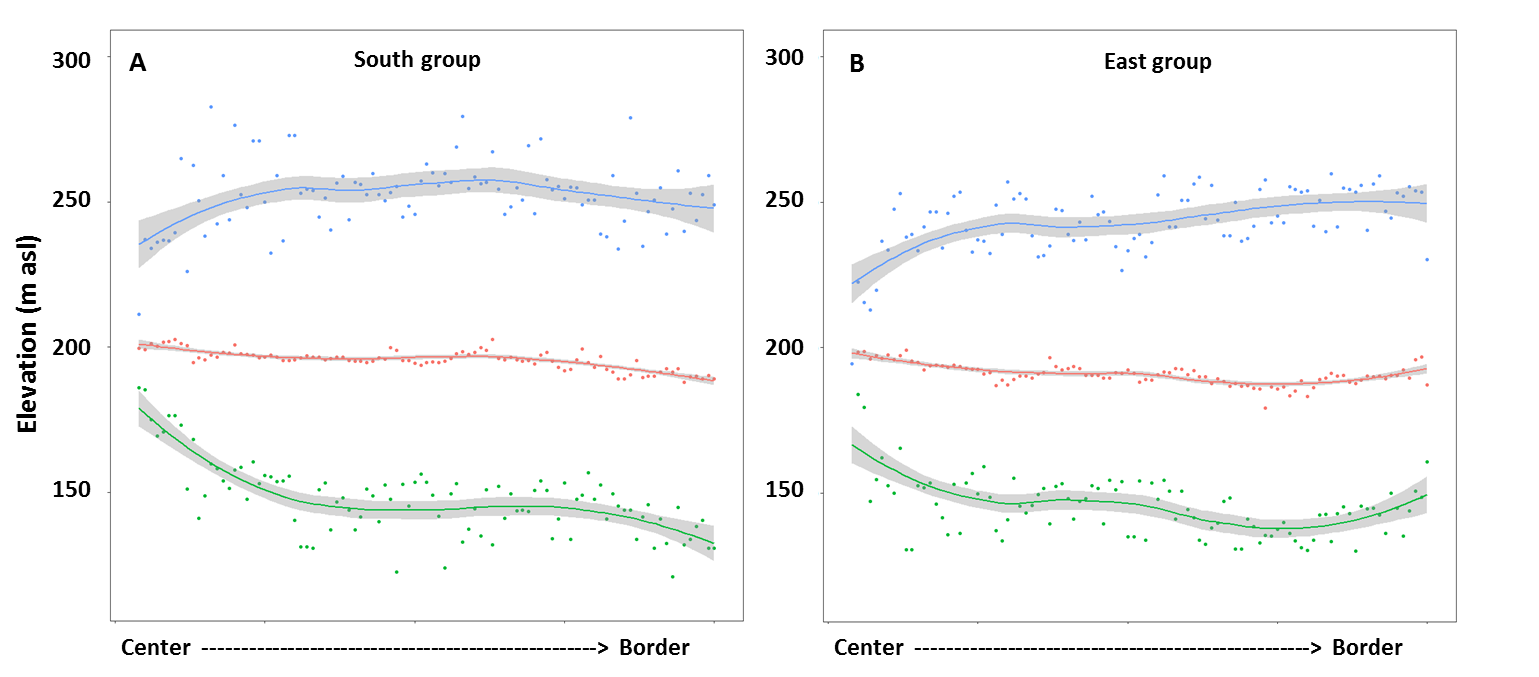

Supplement: S2 Fig — (DOCX) [file pbio.3002350.s011.docx]
