# Supplementary material for: Chimpanzees make tactical use of high elevation in territorial contexts
Source: PLoS Biol. 2023 Nov 2;21(11):e3002350. doi: 10.1371/journal.pbio.3002350 (PMC10621857; doi:10.1371/journal.pbio.3002350)

**S3 Fig**. **Comparison of the average elevation per kernel value between the core area (kernel values below 75) and the periphery (kernel values above or equal to 75), across the entire territories of South (A) and East (B) groups.** Thick lines represent the median, intermediate lines the 25^th^ and 75^th^ percentile and top and bottom grey lines the 97.5^th^ percentile and 2.5^th^ percentile respectively. The raw data underlying this Figure may be found in S2 Data.


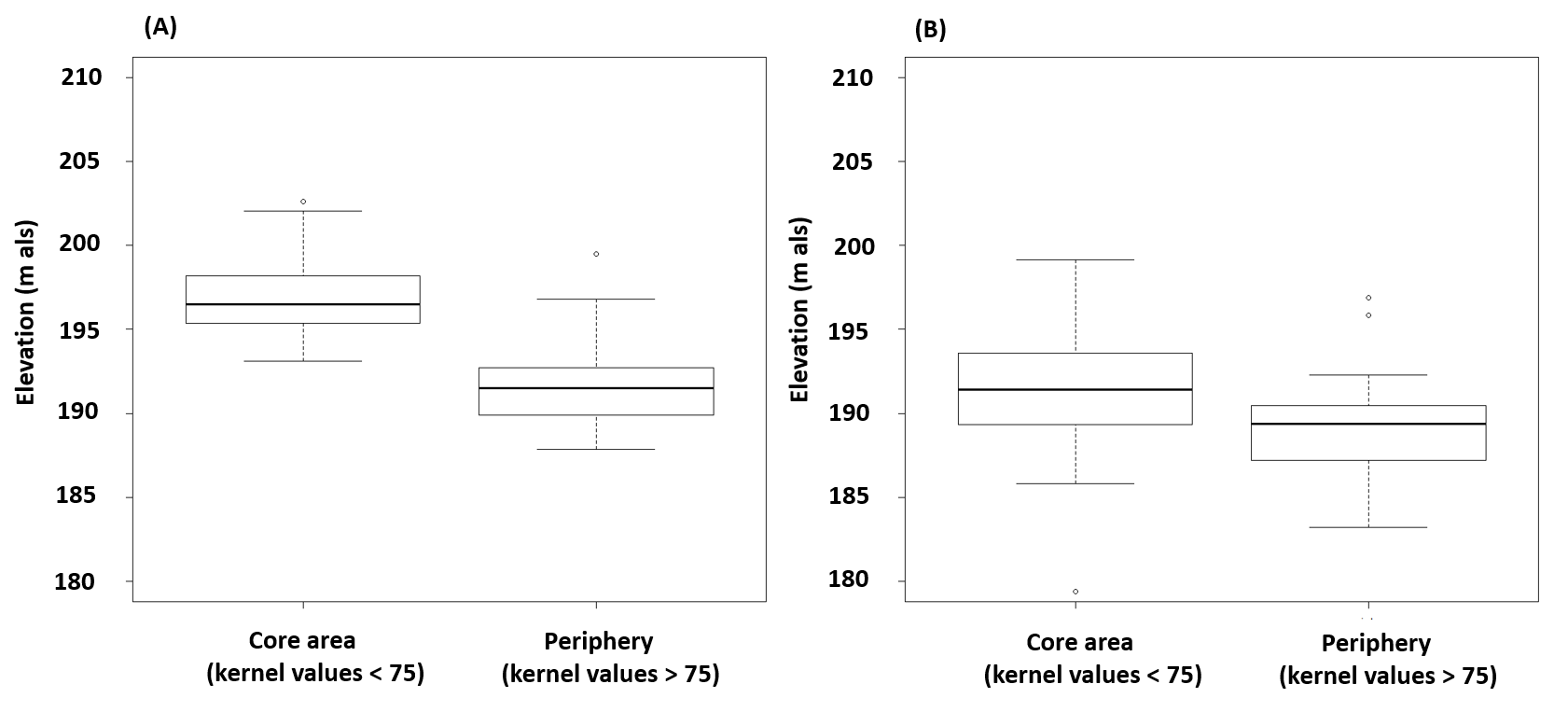

Supplement: S3 Fig — (DOCX) [file pbio.3002350.s012.docx]
