# Supplementary material for: Chimpanzees make tactical use of high elevation in territorial contexts
Source: PLoS Biol. 2023 Nov 2;21(11):e3002350. doi: 10.1371/journal.pbio.3002350 (PMC10621857; doi:10.1371/journal.pbio.3002350)

**S4 Fig.** **Distributions of the used elevation, across the whole territory, for each activity (resting, feeding, traveling), for both South and East group together.** Distributions are split between four territorial categories: core area (kernel values < 25), post-core area (kernel values 25-50), pre-periphery (kernel values 50-75), periphery (kernel values > 75). The raw data underlying this Figure may be found in S2 Data.


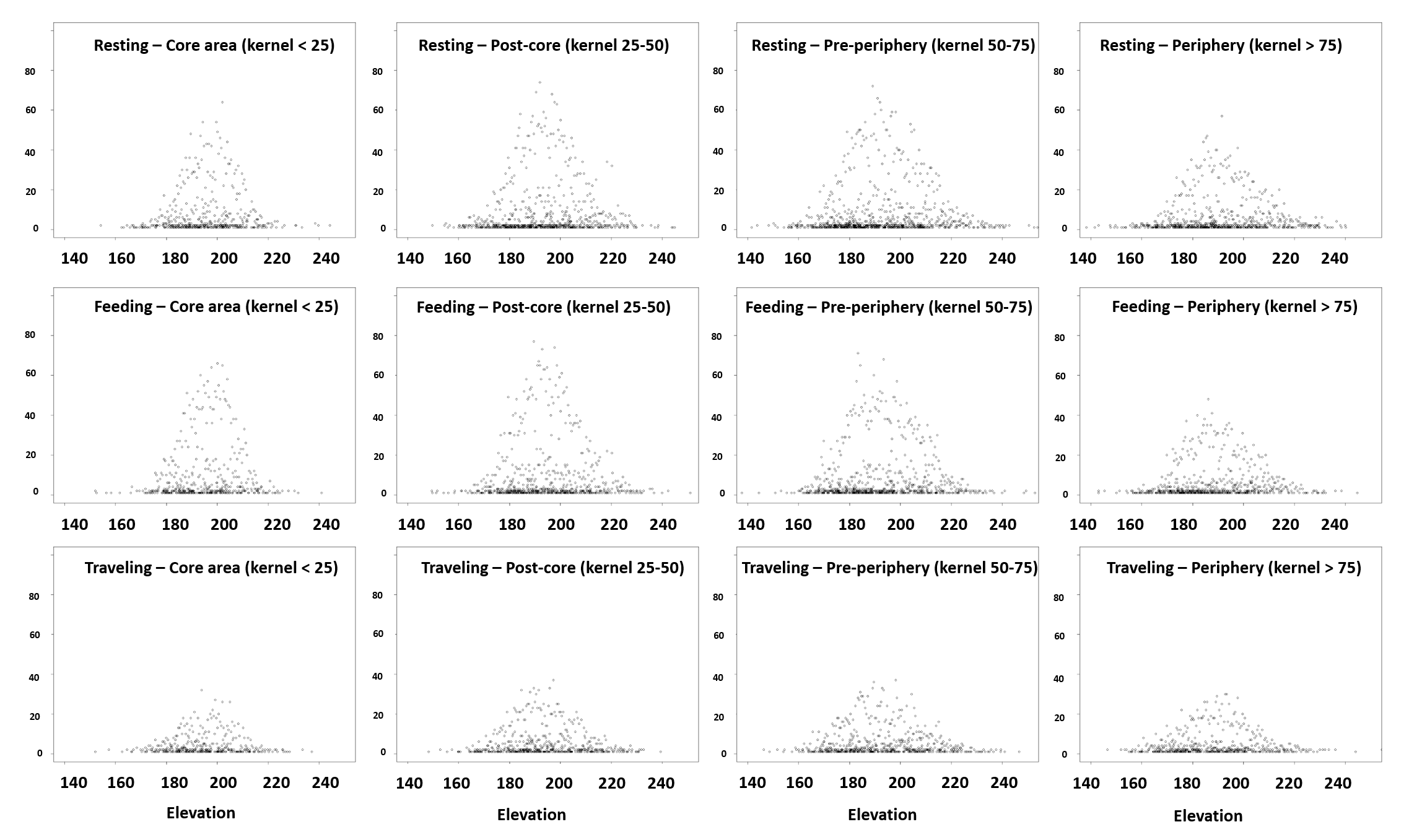

Supplement: S4 Fig — Distributions are split between 4 territorial categories: core area (kernel values <25), post-core area (kernel values 25–50), pre-periphery (kernel values 50–75), periphery (kernel values >75). (DOCX) [file pbio.3002350.s013.docx]
