# Supplementary material for: Chimpanzees make tactical use of high elevation in territorial contexts
Source: PLoS Biol. 2023 Nov 2;21(11):e3002350. doi: 10.1371/journal.pbio.3002350 (PMC10621857; doi:10.1371/journal.pbio.3002350)
Supplement: Attachment — Submitted filename: response to reviewers_clean.docx [file pbio.3002350.s021.docx]

*Dear editors,*

*We first would like to thank the reviewers for their time and for their fruitful and productive comments and suggestions, which greatly improved the quality of our paper. To address concerns that the paper was too dense with many models, and that elevation was not tested within a single model, we have thought carefully about ways to simplify the paper whilst maintaining the integrity of the analyses. As a result, some substantial changes had to be brought. In the original manuscript, we tested, with a four-way interaction, the combined effect of imbalance of power (measured by 2 continuous variables – own adult party size and rivals adult party size), inter-community distance and rivals’ movement. Given the reviewers suggestion to test the effect of elevation, adding a 5^th^ variable (elevation) to this interaction would complexify the analysis, as 5- way interactions are extremely hard to interpret. To accommodate the critical variable of elevation, we thus needed to simplify the model in other ways, which we feel overall adds clarity to the results – and we thank the reviewers for making this suggestion. Hence, we made the additional adjustments: 1) we measured imbalance of power as the arithmetic difference between own adult party size and rivals adult party size. 2) prioritising rival distance, we removed the variable movement of rivals. With the aim of simplifying the paper, we kept one set of analyses in this section (model on the likelihood to advance/retreat), and we removed the models on the distance travelled, given that results were similar and hence contributed little additional information. In the new analysis, we now test a more interpretable 3-way interaction between imbalance of power, elevation and inter-community distance. We also provide a graphical representation of our expectations regarding this interaction (Fig 1). In all, we feel these changes really improve the clarity of the paper, as well as the interpretation of the results.*

*We have also addressed the reviewers’ comments regarding the implications of our results concerning cognitive skills and processes of metacognition, which now deserve their own section in the discussion. We have also modified headings and added sub-headings to help the reader throughout the text. We have been able to address all the suggestions and comments from the reviewers, and we think that this has improved a lot the quality and clarity of our manuscript.*

*You will find our responses to each comment below, marked in italic. Line numbers refer to the new clean version (not the track changes version).*

*Sincerely,*

*Dr Lemoine*

Dear Dr Lemoine,

Thank you for your patience while your manuscript "Tactical use of high elevation in territorial contexts in chimpanzees." was peer-reviewed at PLOS Biology. It has now been evaluated by the PLOS Biology editors, an Academic Editor with relevant expertise, and by four independent reviewers. Please accept my additional apologies for the delay incurred over the busy holiday period.

In light of the reviews, which you will find at the end of this email, we would like to invite you to revise the work to thoroughly address the reviewers' reports.

As you will see below, the reviewers are broadly positive, but each raises a number of concerns that will need to be addressed before further consideration. For example, you’ll see that reviewer #1 wants you to clarify several aspects of their approach, and is uncertain whether you can show that the behaviour at the top of hills is specific to that location. Reviewer #2 is very positive, but questions one of your claims, asks you to consider the meta-cognition literature, and wonders whether the two groups of animals were consistent in their behaviour. Reviewer #3 is also positive, but wonders about two possible alternative explanations (scouting for routes, time-of-day effects); s/he also questions some unexpected behaviour, and asks for several clarifications. Reviewer #4, like the others, is broadly positive, but has difficulties with the density of the manuscript, which makes it hard to follow what the you actually did (several of these concerns, very clearly laid out,

overlap with those raised by the other reviewers); this reviewer also raises problems with your presumptive use of words like “tactical.”

Given the extent of revision needed, we cannot make a decision about publication until we have seen the revised manuscript and your response to the reviewers' comments. Your revised manuscript is likely to be sent for further evaluation by all or a subset of the reviewers.

We expect to receive your revised manuscript within 3 months. Please email us ([plosbiology@plos.org](mailto:plosbiology@plos.org)) if you have any questions or concerns, or would like to request an extension.

At this stage, your manuscript remains formally under active consideration at our journal; please notify us by email if you do not intend to submit a revision so that we may withdraw it.

**IMPORTANT - SUBMITTING YOUR REVISION**

Your revisions should address the specific points made by each reviewer. Please submit the following files along with your revised manuscript:

1. A 'Response to Reviewers' file - this should detail your responses to the editorial requests, present a point-by-point response to all of the reviewers' comments, and indicate the changes made to the manuscript.

*NOTE: In your point-by-point response to the reviewers, please provide the full context of each review. Do not selectively quote paragraphs or sentences to reply to. The entire set of reviewer comments should be present in full and each specific point should be responded to individually, point by point.

You should also cite any additional relevant literature that has been published since the original submission and mention any additional citations in your response.

2. In addition to a clean copy of the manuscript, please also upload a 'track-changes' version of your manuscript that specifies the edits made. This should be uploaded as a "Revised Article with Changes Highlighted" file type.

*Re-submission Checklist*

When you are ready to resubmit your revised manuscript, please refer to this re-submission checklist: <https://plos.io/Biology_Checklist>

To submit a revised version of your manuscript, please go to <https://www.editorialmanager.com/pbiology/>  and log in as an Author. Click the link labelled 'Submissions Needing Revision' where you will find your submission record.

Please make sure to read the following important policies and guidelines while preparing your revision:

*Published Peer Review*

Please note while forming your response, if your article is accepted, you may have the opportunity to make the peer review history publicly available. The record will include editor decision letters (with reviews) and your responses to reviewer comments. If eligible, we will contact you to opt in or out. Please see here for more details:

<https://blogs.plos.org/plos/2019/05/plos-journals-now-open-for-published-peer-review/>

*PLOS Data Policy*

Please note that as a condition of publication PLOS' data policy (<http://journals.plos.org/plosbiology/s/data-availability>) requires that you make available all data used to draw the conclusions arrived at in your manuscript. If you have not already done so, you must include any data used in your manuscript either in appropriate repositories, within the body of the manuscript, or as supporting information (N.B. this includes any numerical values that were used to generate graphs, histograms etc.). For an example see here: <http://www.plosbiology.org/article/info%3Adoi%2F10.1371%2Fjournal.pbio.1001908#s5>

*Blot and Gel Data Policy*

We require the original, uncropped and minimally adjusted images supporting all blot and gel results reported in an article's figures or Supporting Information files. We will require these files before a manuscript can be accepted so please prepare them now, if you have not already uploaded them. Please carefully read our guidelines for how to prepare and upload this data: <https://journals.plos.org/plosbiology/s/figures#loc-blot-and-gel-reporting-requirements>

*Protocols deposition*

To enhance the reproducibility of your results, we recommend that if applicable you deposit your laboratory protocols in protocols.io, where a protocol can be assigned its own identifier (DOI) such that it can be cited independently in the future. Additionally, PLOS ONE offers an option for publishing peer-reviewed Lab Protocol articles, which describe protocols hosted on protocols.io. Read more information on sharing protocols at <https://plos.org/protocols?utm_medium=editorial-email&utm_source=authorletters&utm_campaign=protocols>

Thank you again for your submission to our journal. We hope that our editorial process has been constructive thus far, and we welcome your feedback at any time. Please don't hesitate to contact us if you have any questions or comments.

Sincerely,

Roli Roberts

Roland Roberts PhD

Senior Editor

PLOS Biology

[rroberts@plos.org](mailto:rroberts@plos.org)

------------------------------------

REVIEWERS' COMMENTS:

Reviewer #1:

This is an impressive data set from two neighbouring communities of wild chimpanzees that has been thoughtfully analysed to address interesting questions regarding the use of high elevation by chimpanzees in border areas to gather information on rivals that can be used to minimise the risk of disadvantageous inter-community encounters.

I have a number of suggestions which focus on making the paper easier to read and follow for the reader and a few analysis queries. There were a few places where I didn't understand the approach that was being taken to address a question, that raised concerns that were later allayed when details of all parts of the analysis were provided - please try and ensure you give the reader an accurate overview of how you are going to tackle your questions to prevent other readers having this experience!

L82 Spelling - assess not asses

*This has been changed (L87)*

L96-108- I did not find this a good summary of the model run to test this prediction. Before I had read the results I commented "I don't understand the rationale behind this comparison. It is well known that chimpanzes behave cryptically in border areas (less likely to feed, frequent stops/rests to listen) - so surely this is what you would expect comparing behaviour from a central to a periphery area regardless of elevation. I would like to see an elevated duration of resting in high vs low elevation locations in border areas to be convinced that they are resting to listen / monitor the neighbours". Please try and make it clearer that you test for an interaction between elevation and border/central location to test your prediction

*We acknowledge that it is well known that activities change as function of the location within the territory, and we refer to this in several instances. What is not known is whether activities also change as function of the used elevation. To test the elevation hypothesis, we tested the interaction between elevation and location in this activity analysis. The interaction allows to account for both effects and to capture contrasts in activity as function of elevation in different parts of the territory We also now clarified in the text that we test the effect of the interaction between location and elevation and we added a statement justifying the test of this interaction (L108-111).*

*We also provide a table (Table S2) of the percentages of time spent in each activity in four sections of the territory (periphery defined as kernel values above 75; kernel values between 50 and 75 (pre-periphery); kernel values between 25 and 50 (past core) and kernel values below 25 (core), for both groups and between groups). We provide, in S4 Fig, distributions of elevation used for each activity, across these 4 sections of the territory. We present this table in the result section (L213-217) and S4 Fig L239-242). Activity budgets confirm an increase of resting and traveling in the periphery as compared to the core area. Distributions of used elevations show that resting at high elevation in the periphery occurs more often than resting at high elevation in the core. It also shows that lower elevations are more used in the periphery as compared to higher elevations (reflecting the relative distribution of high and low elevation across the landscape). In parallel, we find that resting at high elevation in the border is more likely than at same high elevation in the center, despite spending more time resting at lower elevations in the border. We interpret this contrast as a preference for resting at high peripheral elevations, as compared to the center of the territory.*

*A side note on the fact that chimpanzees spent more time resting at low elevation than at high elevation. One could argue that this pattern contradicts our hypothesis of high elevation territorial usage, where one could expect that a risk reduction strategy should lead to preference to use high elevation to rest in the periphery. However, landscape is heterogeneous and higher hills and high elevation only represent a fraction of all elevations. Thus, this differential in usage of elevation in the periphery is likely due to the structure of the landscape, which is lower in the periphery than in the center for both groups. In contrast, our key finding is that high peripheral elevations are more likely to be used for resting than similarly high elevation in the center, while central areas are generally higher than peripheral ones. This is compatible with a territorial usage of high elevation in the periphery.*

L171-80 - unusual to summarise the results in the intro - I would remove this unless it's a journal requirement

*We follow your advice and have removed that section, also making the text less dense.*

L254: Hill top information determines advances towards rivals

In this model you only include occasions where the chimps stopped on the hills. How do you know the behaviour is specific to having stopped on the hills unless you compare it to instances when they do not stop on hills

I have now read on and seen you run a separate model for stops on high hills and low hills. I am confused as to why you did not run a single model with these 2 data sets to test for an interaction between stop elevation and rival information. I think this is what you need to show that rival information more likely influenced decisions when stops were on high elevations.

*Following your suggestion, we have now combined both datasets and tested the effect of elevation in a single model. As compared to our previous manuscript, quite a few changes had to be brought, to reduce the length and density of the paper and to not overcomplexify the analyses. In the original manuscript, we tested, with a four-way interaction, the combined effect of imbalance of power (measured by 2 continuous variables – own adult party size and rivals adult party size), inter-community distance and rivals’ movement. Adding a 5^th^ variable (elevation) to this interaction complexifies the analysis as 5- way interactions are extremely hard to interpret. To accommodate the critical variable of elevation, we thus needed to simplify the model in other ways, which we feel overall adds clarity to the results – and we thank the reviewers for making this suggestion (L254-279). Hence, we made the additional adjustments: 1) we measured imbalance of power as the arithmetic difference between own adult party size and rivals adult party size. 2) prioritising rival distance, we removed the variable movement of rivals. With the aim of simplifying the paper, we kept one set of analyses in this section (model on the likelihood to advance/retreat), and we removed the models on the distance travelled, given that results were similar and hence contributed little additional information. In the new analysis, we now test a more interpretable 3-way interaction between imbalance of power, elevation and inter-community distance. We also provide a graphical representation of our expectations regarding this interaction (Fig 1). In all, we feel these changes really improve the clarity of the paper, as well as the interpretation of the results. Overall, results point toward an advantage of higher elevation in term of rival detection of presence or absence.*

L335-337 / 387-388 - The summaries of this finding matches the intro, but doesn't seem to capture the crucial interaction in the model that showed that rest was more likely at high elevation in border areas rather than central high areas or low border areas.

*We have changed the formulation of these sentences, to emphasize on this key finding (L314-316 and L343-345).*

L364 - you don't present data on intentional use of this information so I would remove this claim

*We have removed the word intentional (L331). To address comments by other reviewers we have added a section on the implications of the results for interpreting cognitive function (see below) (L404-463). Of note, we address this extremely carefully, noting in several places that a behavioural study under natural conditions, such as this, can identify paradigms that may engage complex cognition and therefore be suitable for designing related cognitive tests.*

Discussion - I think this could be improved by shortening and focussing on interpretation of the results. There are quite a few more speculative paragraphs (e.g. on social cognition and selection pressures) that I think could be removed, and careful editing to remove repetition and more tangential points would really improve this. Some parts read as if they have been inserted to satisfy reviewer critiques, but often the potential issue they counteract is not very well explained, making the relevance of these parts less clear and slightly random to the reader.

*We have followed this advice, in removing and shortening some parts, removing repetitions and in re-structuring our discussion. We have also added subtitles to make the discussion clearer and easier to follow. However, given the complexity of analyses and results, but also their implications, we think that a thorough discussion on the results is necessary, as well as expanding on the implications of our findings regarding cognition and human evolution – a request of reviewer #2. However, we hear the points raised by each reviewer relating to the cognition and hence have formulated these with particular care, aiming to remove more speculative statements. Specifically, we do not claim to test cognition but point out that the results are consistent with certain cognition capacities, such that formal cognitive testing would be warranted. We also tempered our statements regarding how intergroup competition could constitute a selective pressure and territorial topography usage. We hope that this restructuring makes our paper easier to read and is more convincing.*

L616 - of more than 50m - should be for more than 50m

*This has been changed into “for more than 50m” (L615)*

Reviewer #2:

The authors present evidence of tactical use of high elevation locations by chimpanzees at the periphery of the territory. The authors argue that higher elevations allow for gathering better auditory information about neighbouring (rival) chimpanzee groups and that chimpanzees use to their advantage in intergroup competition (warfare).  The authors use behavioural and environmental data of two neighbouring chimpanzee groups in the Taï National Park, Côte d'Ivoire, across several years. Their main arguments for tactical use of high elevations are that i) chimpanzees are more likely to rest at higher elevations (allowing for information gathering) on the way towards a potential intergroup encounter ii) that chimpanzees do use information gathered about the neighbouring group (e.g. size, distance) to make tactically sensible decisions about their own iii) that they do not show these responses to information about neighbouring groups after resting periods at lower elevations.

I think this is a great, and unique addition to our understanding of intergroup interactions. We get further insight into chimpanzee decision making and, as the authors argue, it also has implications for understanding the role of conflict in hominin evolution. Overall, the analysis seems sound (with the caveat that I am not an expert in using GPS data) and the conclusions supported by the results. There are some slight inconsistencies in how strongly the authors interpret the tactical use of higher elevations (i.e. that chimpanzees move to a higher elevation intentionally with the purpose of getting information on neighbouring groups) but I think these are relatively minor. The majority of my comments are suggestions are cosmetic rather than fundamental to the manuscript and are listed below:

Interpreting intentionality of elevation use

The authors very accurately state their aims to empirically test for "behavioural patterns are consistent with those expected given tactical decisions that result in minimizing risks" (L53-54). The evidence presented does support this claim, which is a significant addition to the literature. There is good evidence that they are responding to information about the rival group. I think it is more difficult to argue for the intention of spending time at elevation to gather information on rival group activities with the data presented here and the general complexity of observational data in natural environments where we know there are a large number of factors that are likely to involved in individual decision making at any one point. The authors do briefly acknowledge that there are potentially alternative explanations for resting pattern observed (Line 379). However, this feels imbalanced in comparison to the author's argument that "it is likely that hill climbing for

information-gathering is done intentionally" (Line 384) and another similarly strong statement on line 485.

*The reviewer suggests that our results do not necessarily demonstrate intentional use of hills to detect rivals, but could be a by-product of other behaviours/intentions. We have therefore tempered our strong statements by removing mentions of intentionality, and we acknowledge that our analyses do not explicitly test for intentional territorial use of topography. In the paragraph L361-370, we discuss other possible alternatives than a territorial use of high elevation. In paragraph L426-433, we state that the behavioural patterns observed fit with predictions expected if chimpanzees were engaging in goal-directed and planned behaviour. We also discuss alternative explanations and conclude that these are hard to justify. However, we are careful to assert that formal cognitive testing would be required to confirm specific cognitive capacities.*

The cognitive abilities related to tactical use of elevation

The authors suggest that the ability to take the perspective of what others can see or hear demonstrated in behavioural experiments supports their claim that chimpanzees could be using the elevation strategically. Could I suggest that the metacognition, especially information-seeking, literature would be more appropriate for interpreting the behaviour being studied here? It aims to understand how animals modify their search behaviour in response to their own knowledge (or lack thereof) as well as the quality of potential information sources. I think the current results would also be of a lot of interest to researchers in this area. I have suggested a short review that summarises the evidence for information seeking and highlights key papers:

Marsh, H. L. (2019). The information-seeking paradigm: Moving beyond 'if and when' to 'what, where, and how.' Animal Behavior and Cognition, 6(4), 329-334. <https://doi.org/10.26451/abc.06.04.11.2019>

*We thank the reviewer for this useful comment, of which inclusion makes our paper stronger and more pertinent. We now offer this framework in the discussion as a possible way to interpret our results and from which formal cognitive testing would likely be insightful. Given that this was not an initial hypothesis, nor directly tested, we did not include this in the introduction.*

*We added elements of metacognition and what our results suggest regarding this aspect in its specific section on cognitive implications of our findings (L404-463), and specifically L434-454.*

The last section of the discussion (L494-507) lists a number of areas of cognition that could be involved in decision-making in this context. To avoid it just being a list, it would be good to have some expansion on specifically how using higher elevation could be used to study these areas of cognition.

*We have now included a section on implications of our findings regarding cognition, which we think is now more balanced and thorough. (L404-463).*

Group differences

Out of interest, were the patterns of decisions consistent between the two groups studied? I see from the models that this was not formally tested, that group membership was a control. I assume this was to reduce model complexity - given that there were already 4-way interactions involved - but I am curious as to whether the strategy was group specific or observed in at least these two groups.

*As the reviewer points out, we have not formally tested group differences. First, we did not have a specific prediction as to why there would be group differences and second, adding group as an interaction would again make the models too complex. Only having group as a control reveals that in most analyses, especially those on movements from high elevation after gathering information from rivals, groups do not differ in the responses.*

Minor Points

Line 36-38 This sentence is quite difficult to parse. Maybe it could be re-ordered or re-worded to make it easier to interpret. There are a few other very long, complex sentences with multiple subclauses dotted throughout the manuscript that could also benefit from simplification.

Line 45-47 See previous point

*We have modified the structure of these long sentences in several locations throughout the entire manuscript.*

Line 110 Word missing: "Riskier situations to the in-group"

*Change added L124.*

Line 124 The clarity of Table 1 could be improved. Some suggestions: only have "Risk minimization movements" once; remove the parentheses, change to text for quicker interpretation ('advancing increases risk', instead of just "increased risk".

*We have now replaced that table with a graphical representation of the expectations on the tested 3-way interactions between imbalance of power, inter-community distance and elevation on the likelihood to advance towards rivals (Fig 1). Predictions regarding the stop model are embedded into the text (L97-100).*

Line 174-180 Could this sentence be broken up?

*This paragraph has been removed, following another reviewer’s suggestion. A summary of results at the end of the introduction wasn’t necessary.*

Line 384 The authors suggest there are multiple studies supporting their statement but only cite one

*That sentence has been changed and moved further down (L426-433), and several other references have been added regarding chimpanzee abilities of perspective taking (ref 58-62)*

Line 412-414 Could the authors expand on the reasoning here. It's not clear exactly how this fits together.

*That part has been removed as it does not fit within the content. The original idea behind this statement is that, given other studies show that coordination is high during territorial activity, all members from a given party most likely adopt the same traveling decisions and remain coordinated.*

Line 453-454 I think this is very interesting, these contexts could provide a stronger test of the intentional information seeking hypothesis.

*Thanks for pointing this out. We have kept a statement in this direction L368-370.*

Supplementary Information, S1A Table

Typo 1: Variable given different names as a main and interaction effect ("own party size" and "adult party size")

Typo 2: "(b) test predictors, in bold" - test predictors not in bold

*Changes have been made in the appropriate places.*

Reviewer #3:

In this manuscript, the authors investigate an exciting, creative, and - to my knowledge - novel question: do humans' closest chimpanzee relatives exhibit tactical use of high elevation for information-gathering in intergroup contexts? This work is likely to be of high interest, with relevance to behavioral ecology, human evolution, and psychology. It is also possible only because the authors have access to a unique field site where they have habituated, and collect concurrent data on, multiple neighboring chimpanzee communities. The authors address their question with a number of thoughtful, well-reasoned analyses. I am very much in support of publishing this work and offer several points for the authors' revision.

The body of results are consistent with the authors' interpretation but two additional alternative explanations - at least for visitation of hilltops - come to mind:

(1) when traveling outside of their familiar core area, chimpanzees may seek vantage points to determine the appropriate route (and/or identify feeding locations), not just to survey rivals. Such scouting may not be necessary when returning to core areas.

(2) The tendency to visit/rest at hilltops may be influenced by the time of day, with animals tending to move more quickly and efficiently (avoiding hilltops and/or rest) at later times of day when they are returning to their nesting site before dark (and/or because it has already become dark and little vantage is offered by hilltops), than at earlier hours of day when they have time for leisure and/or better conditions for perception. Chimpanzees might have fewer stop events on peripheral hills on their return to the territory center because these occur later in the day, when visibility is worse and/or when chimpanzees are motivated to more quickly get back to their nesting site.

*Thank you to the reviewer for these constructive comments. Following these comments, and other reviewers’ similar comments, we have brought some nuance in our interpretation of intentional high hill usage, solely for territorial purpose (L361-370). Indeed, it is possible that detecting rivals from high spots may just be a by-product of other non-territorial behaviour, such as searching for resources, raised sunny spots, and indeed landmark usage while traveling. We also discuss the reasons why these alternatives are unlikely to explain the observed patterns of behaviour. We also note that whilst our analyses do not explicitly test for goal-oriented use of high peripheral elevation, our results are consistent with the predicted pattern of the “what, where and how”. We also discuss goal-oriented behaviour and potential for intentionality in paragraph L426-433.*

*Climbing and stopping at hills was indeed more likely early in the day, as it was more likely on the way towards the periphery. In addition, traveling towards the periphery is more likely early in the day (see results L197). We then interpret the effect of time of the day as a confound effect with their traveling toward the periphery, which most of the time occurs early in the day. This effect does not rule out the fact that stopping on the way towards the periphery can be part of territorial tactics.*

If the authors have data on movement out of core areas to non-peripheral areas (e.g., foraging trips), it would be possible to compare visitation of hilltops during foraging versus patrol events, controlling for time of day. The tactical elevation hypothesis predicts that the direction of travel should determine visitation of hilltops, independent of time of day. If tactical use of elevation is specific to intergroup monitoring, it should be observed more on journeys to patrol the periphery than on journeys to feed in non-peripheral, non-core areas. In the event that such metrics are not easily available and the authors are unable to pursue this additional analysis, I would still advocate the paper's publication. However, if the authors cannot exclude these alternative explanations with additional analyses, the explanations should be discussed in the text. The existing finding that time of day is related to hilltop visitations certainly warrants more discussion.

*To our own view, it does not matter whether they are engaging in patrols to encounter rivals or to raid food. Since one can lead to the other (ie raiding food from other's territory is highly risky leaving one vulnerable to ambush), similar tactics should be employed in both scenarios, hence here we do not distinguish them here.*

*Also, patrols may be triggered to detect rivals, but also in response to rivals already been detected (in which case we would expect less usage of high areas). Disentangling both situations (response to detection, or aim for detection during patrols) would be difficult at this stage. In addition, core area and periphery are exclusive, but there is no third part of the territory. Looking at feeding trips within the core area compared to feeding trips in the periphery is already tested in the activity analysis. Looking at feeding trips into the periphery and comparing it with patrols into the periphery would be an option, however, as said, patrol may be started in the knowledge of the whereabouts of the rivals, which would not require the need to visit many trees. However, we acknowledge that further analyses of the link between landscape use and territorial events would warrant more focus, especially to fully determine a territorial intentionality behind elevation use (L368-369).*

In my view, the weakest findings (or those that conform least clearly to the authors' hypotheses and interpretations) are presented in lines 255-278. One set of findings is predicted and the other is not: When rivals are retreating or staying put, chimpanzees respond in ways that are consistent with potentially comparing party numbers (advancing when own number are higher and when rival numbers are lower). However, when rivals are advancing, chimpanzees respond in unexpected ways (advancing when own numbers are lower and when rival numbers are higher). If chimpanzees did not detect their rivals during rivals' (potentially quiet) approaches, as the authors suggest, there should be no effect of own number or rival number. Inspection of the graphs suggests that this might actually be the case, at least for the own party number finding. It would be useful to see more discussion of this, and of any reasons why chimpanzees might be more likely to approach larger groups of rivals.

Is this somehow an artifact of the models/interactions? Given the slightly mixed support for information-gathering in this section, it may also be worth changing the header to be more neutral (i.e., to not imply a claim of information-gathering). That said, I do acknowledge that the bulk of the results are in line with information-gathering, and that this body of findings is informative and interesting and add value to the manuscript.

*We acknowledge that this was a puzzling result. It is possible that these result from the complexity of the interaction, even if our data are well distributed across the variables involved in the interaction. Following another reviewer’s suggestion, we have now combined both datasets and tested the effect of elevation. As compared to our previous manuscript, quite a few changes had to be brought, to reduce the length and density of the paper and to not overcomplexify the analyses (please see above the response to reviewer#1). In order to avoid testing a five-way interaction (former four-way interaction * elevation), which would have been extremely difficult to interpret, we simplified the analysis. The new model is testing the effect of the interaction of the imbalance of power, elevation and inter-community distance, providing an analysis to test the predictions of tactical elevation use (see Fig 1). We are confident that these changes improve the clarity of the paper, as well as the interpretation of the results.*

Because chimpanzees live in fission fusion societies, at any given time a chimpanzee community may be comprised of a number of different parties in various areas of the territory. Which subject and rival parties were chosen for these analyses? Do the data about the rival party refer to the rival party that is closest to the subject's party at the time of data collection? These critical details should be clarified in the main text (not just the methods).

*We have added this clarification in the introduction (L155-156), by saying that, given the fission-fusion nature of chimpanzee societies, we considered as inter-community distance the distance of the closest recorded party to the focal group using a specific location.*

*We followed each chimpanzee community each day with between 1-3 observers following different target chimpanzees. Focal chimpanzees sometimes separate into several parties. The closer parties approach the periphery, the more likely they fusion. When we followed several parties this day, we considered the closest party to the focal party for the inter-community distance. As stated now in the methods (L598-601), it is important to note that in Tai chimpanzees, communities are considerably more cohesive than in other populations such that adults spent less than 5% of their time alone, and between 45 and 65% of their time in mixed sex parties (Lemoine et al., 2019) or within 1km of each other (Eckhardt et al, 2015).*

*However, although very unlikely, we cannot guarantee that there were not undetected parties around, either in the focal community nor in the rival community. These statements are now provided L598-601.*

It would be useful to have at least an anecdotal description of whether hilltops provide a better visual vantage point (e.g., less vegetation) or are only thought to enable clearer perception of auditory information.

*We have added a brief statement about this aspect in the discussion (L363-365), as well a reference (Piña-Covarrubias, E., Hill, A.P., Prince, P., Snaddon, J.L., Rogers, A. and Doncaster, C.P. (2019), Optimization of sensor deployment for acoustic detection and localization in terrestrial environments. Remote Sens Ecol Conserv, 5: 180-192). Also, from our experience, most of high hills do not offer a particular good point of view due to the dense vegetation covering them, but they clearly improve acoustic conditions (experienced by hearing gunshots much more clearly).*

The article is well written. However, many sentences read as relatively dense because they span multiple lines of text and sometimes include more than two clauses (or a large number of comma-separated units of text). The manuscript's readability could be improved in such cases by breaking these segments into multiple sentences.

*Following this comment and other reviewers’ similar comments, we did our best to break down long sentences and make the reading more flowing, throughout the entire manuscript.*

Line 191 - when reading the main text, it is not immediately clear whether the dependent measure is all instances where chimpanzees were in the vicinity of a peripheral hill and whether (1) or not (0) they stopped, or all instances where chimpanzees climbed a peripheral hill and whether (1) or not (0) they stopped, or something else. Since the casual reader will focus on the main text, it would be helpful to make this point clearer within that part of the manuscript. I believe it is the second of these options; slight changes in wording would greatly improve clarity (e.g., changing 'all occurrences of peripheral hill use' to 'all instances in which chimpanzees climbed a peripheral hill' AND 'patterns of stop events' to 'the presence or absence of stop events').

*We have made these proposed clarifications (L183-187), by saying “**To analyze whether chimpanzees prioritize stop events on peripheral hills, we used a GLMM with binomial error structure* ***on*** ***all instances in which chimpanzees climbed a peripheral hill*** *(n = 717 occurrences). Specifically, we analyzed* *whether the* ***presence (coded as 1) or absence (coded as 0) of stop events*** *on peripheral hills (≥ 5 min of resting)* *is influenced by the previous movement direction, by the interaction between own adult party size and inter-community distance, and by other peripheral hills use.”*

Line 291 - if the metric is distance of advance, why is the header speed of advance?

*The idea behind speed was that the extent of movement (in meters) is standardized within the 30min following departure, corresponding then to a distance related to a time slot. However, in a bid to simplify the analyses and text in the paper (a request from several reviewers), and because this adds little given a similar analysis on the likelihood to advance towards rivals, we have removed this.*

All data appear to be present but would be enriched by inclusion of code (e.g., R scripts) to replicate the authors' analyses

*R scripts are now provided, along with the datasets used in analyses.*

Reviewer #4:

In this paper, Lemoine and colleagues explore the way in which chimpanzees at Tai National Park changed their behavior after resting for 5 min or more on the exposed hilltops in the borders of their territory, especially those borders that overlapped with other groups' territories, with the assumption that their behavior changed due to information gained about the location and size of parties of chimpanzees from other groups.  I am enthusiastic about this paper, as I appreciate both the use of a long term data set to answer a really difficult question and their interest in relating it to an important question in human evolution.  On the whole, I think that with some edits it will be a good fit in PLOS Biology.  That being said, I have some concerns about the analyses and writing, which I address below.

One challenge I had with the paper was figuring out exactly what the authors did.  The paper is extremely dense with predictions and analyses and repetitive in places, which made it difficult to follow; I am not certain that I understood their analyses.  However, it appears to me that the major findings were based on an analysis of the way chimpanzees behaved when they were on peripheral vs core hills.  But to me, a key factor is how they respond when they are on hills vs NOT on hills.

*Thank you to the reviewer for these constructive comments. Following similar comments from other reviewers, we are now testing the effect of elevation on the likelihood to advance towards rivals’ location in a single model (please see details above, in responses to reviewer#1). In order to avoid testing a five-way interaction (former four-way interaction * elevation), which would have been extremely difficult to interpret, we simplified the analysis. The new model is testing the effect of the interaction of the imbalance of power, elevation and inter-community distance, providing an analysis to test the predictions of tactical elevation use (see Fig 1). We are confident that these changes improve the clarity of the paper, as well as the interpretation of the results.*

Of course, I may have misunderstood, so I have outlined my understanding of the results below, so if I misunderstood the authors can use it to know how to clarify and otherwise they can use it as a suggestion for additional analyses.  Looking at each section, I see that:

* Chimps stopped more in hills traveling towards the border than towards the core of their territory, and they were less likely to stop on peripheral hills if they were going to be on peripheral hills later in the day (or maybe they were more likely to stop if they hadn't earlier?  Not sure if they can discern causality from this) or if it was later in the day.

*We have changed our statement to make it clearer (L193-194).* *Stopping at a given peripheral hill was less likely when more peripheral hills were subsequently climbed by that day.*

* Resting and travel were more likely at high elevation near the border than near the center, which contrasts with feeding, which was more likely at the center. The latter could be that they were more relaxed at the center, but this contrasts with resting, which they shouldn't do if they aren't relaxed unless there is another purpose.  However, the graphs in Fig 3 look like the effects are rather modest, so I would like to see some effect sizes on these results.  I would also like to know how often they rested on hills in the periphery vs lower areas in the periphery.  If they are resting to listen equally often anywhere in the border then they may be using information about rivals tactically, but they aren't using hills tactically, which is different.

*To clarify, this analysis uses elevation as a continuous variable, so is not looking at hills versus low-lying areas as it is the case in subsequent analyses.*

*We are also providing a table (Table S2) of the percentages of time spent in each activity in four sections of the territory (periphery defined as kernel values above 75; kernel values between 50 and 75 (pre-periphery); kernel values between 25 and 50 (past core) and kernel values below 25 (core), for both groups and between groups). We provide, in S4 Fig, distributions of elevation used for each activity, across these 4 sections of the territory. We present this table in the result section (L212-216) and S4 Fig L238-241). Activity budgets confirm an increase of resting and traveling in the periphery as compared to the core area. Distributions of used elevations show that resting at high elevation in the periphery occurs more often than resting at high elevation in the core. It also shows that lower elevations are more used in the periphery as compared to higher elevations, which is reflected in the topography of the landscape. In parallel, we find that resting at high elevation in the border is more likely than at same high elevation in the center, despite spending more time resting at lower elevations in the border. We interpret this contrast as a preference for resting at high peripheral elevations, as compared to the center of the territory.*

*A side note on the fact that chimpanzees spent more time resting at low elevation than at high elevation. One could argue that this pattern contradicts our hypothesis of high elevation territorial usage, where one could expect that a risk reduction strategy should lead to preference to use high elevation to rest in the periphery. However, landscape is heterogeneous and higher hills and high elevation only represent a fraction of all elevations. Thus, this differential in usage of elevation in the periphery is likely due to the structure of the landscape, which is lower in the periphery than in the center for both groups. In contrast, our key finding is that high peripheral elevations are more likely to be used for resting than similarly high elevation in the center, while central areas are generally higher than peripheral ones. This is compatible with a territorial usage of high elevation in the periphery.*

*Effect sizes are now provided the text for the significant predictors within each specific analysis/model.*

* Movements towards rivals' locations were influenced by whether they were heading to the border in the first place, own party size, and rivals' movement direction, plus and interaction between the latter two.  I particularly like the quantification of the chimps' risk assessments here (lines 271-4), but I didn't entirely understand what was going on in lines 273-8.  It sounds like the chimps counterintuitively were more likely to approach if it was a larger rival group heading towards them? The authors appear to say that this was because the chimps were confused or the rivals were silent (lines 277-8), but wouldn't that be the entire point of using the hills for tactical advantage?

*This section has changed due to the changes in analyses, after this manuscript review. We hope as a result, that our results are now more straightforward and easier to follow.*

* Chimps advanced further towards rivals when they were moving towards the border than the core, which is expected as the rivals are presumably at the border.  They were more likely to advance if the rivals were a small, close group and they were large, and more likely to retreat if there was a large group of rivals.  To be honest, it was not entirely clear how this section (291-305) was different than the previous one (255-278).  That could use some more explanation.

*These two sections corresponded to two different analyses: one on the likelihood to advance/retreat toward/from rivals; the second on the distance towards/away from rivals traveled within 30min after departure (then corresponding to an approximation of a speed of advance). Given the changes brought to the analyses and models, we have only kept one set of models (on the likelihood to advance towards rivals’ location).*

* The last section does compare the use of hills vs low elevation areas, and it may be that the information that I have been looking for is here.  If so, then I suggest that this be put in earlier models, or addressed first, as I think it is the critical comparison.  Indeed, what this section (307-324) appears to suggest is that chimpanzees did stop and rest in low elevation areas, but there is not a comparison to tell us how frequent this was relative to time spent in low vs high elevation areas; that is, are they resting more per time spent in hills or low areas?  For instance, what if they're actually resting more in the low areas if they're spending lots of time in the hills, which could suggest that they're going to the hills for safety and they're more nervous in the low areas, hence they are more likely to rest and listen?  I'm not suggesting this is the case, but merely pointing out that without that data, we don't know what they're doing.  I would like to know how often they are resting in the hills vs in the low areas.

*We have now provided a new analysis which combines both datasets (from highest hills, and from lowest peripheral locations) and test the effect of elevation, in a 3-way interaction with imbalance of power and inter-community distances. Results of these models suggest an advantage of high elevation in detecting rivals’ presence/absence, but not in detecting their numbers (even if numbers matter too, but independently from elevation).*

*As for how frequently peripheral hills are used as compared to low-lying areas, the numbers had already been provided in the methods section (L576-585). One can see that hills are used more often in this overlap area, than low-lying locations (which are also more numerous). Mean stopping times at high and low peripheral locations are also provided in the results section (L257-258), and do not differ much. Table S2 and S4 Fig provide also more information on the distribution of used elevation across the territory and time spent in each activity across the territory.*

Second, it seems that their behavior in low elevations isn't all that different from that in the hills; they are moving forward more from border than core areas and the likelihood to advance and the distance to advance are relative to the party size, just as in the hills.  The one difference appears to be that there is no effect of how large the rival's party is or whether they are advancing or retreating. That is potentially the really interesting bit, although effect sizes so that readers can compare relevance.  I suspect that these tables (S5B and S6B) should be in the main text for comparative purposes.

*Given that we modified our approach by using a single model, including both high and low elevation, these earlier findings shall be disregarded. With our new approach, our results are easier to interpret, and still suggest an advantage of high elevation in detecting rival presence or absence, from longer distances. However, assessment of rivals number appear independent from use of high elevation. The table presenting the reduced model on the likelihood to advance towards rivals, with key results, is now presented in the main text (Table 1).*

Also, all of these analyses chose >  5 min as the cut off for a rest period.  How was that time period chosen? That seems like a fairly artificial cutoff; especially if the chimps are nervous and listening, they may pause more briefly to listen and gain information, and sitting still for that long seems risk.  Why not look at a shorter period?

*As a clarification, the analyses testing the effect of location and elevation on activities does not use this 5min cutoff period, but each occurrence of each activity. However, the 5min cutoff period is used in the analyses taking place in the overlap area (likelihood to stop and to advance towards rivals). We acknowledge that choosing 5min cut off is somewhat arbitrary but also reasonable given the average length of all resting bouts (between 11 and 15 min), and given that chimpanzees do not vocalize often. Having a shorter period would then reduce the possibility to detect/hear rivals’ vocalizations, and may be confused with for example, pausing to wait for a conspecific to catch up. We also consider that stopping at least 5min reflects a real stop, rather than decrease of traveling speed to wait for conspecifics (which happens often). We have added explanations L653-657.*

I would also like to know whether they show the same behavior changes after time in the hills when they are not resting, suggesting that just being in the hills provides that information.  That is, are they hearing more just by virtue of being high, and they don't need to stop and rest?  Or are they hearing more by virtue of being close to the border, so they don't need to rest?  Obviously I don't know what the authors' data structure is, so it may not be possible, but I think this would be very informative.

*First, as said in the text, we are not able to provide details on specific resting behaviour (like increased vigilance) – discussed L349-352. The question of whether behaviours upon departure from high hills would be similar without stopping is pertinent. However, given the changes we now brought (with a combined dataset including both high and low elevation), undertaking an analysis on “non-stop” events at both low and high locations is not feasible at this stage, given the structure of the data. Also, we assume that stopping aids in rival detection, as stopping for a certain time increases the chances to detect rivals. Thus, in this paper, we focus on the pertinence of using elevation to improve information gathering, rather on the importance of stopping. We have, however, added a statement (L417-418) regarding this potential caveat that would require further investigation.*

*A statement highlighting the potential caveat of not knowing whether behaviours would be similar without stopping has been added L418-419.*

Finally, these data are highly consistent with what we know of chimpanzee patrols already (they pay attention to their party size and that of their neighbors, and only advance if their party size is the greater), so it would be good to expand upon how what the authors are seeing of their behavior in the hills adds to or changes the understanding we already have.

*One main contribution here, is that not only own numbers and rivals’ numbers are detected and considered to undertake movement manoeuvres, but also how far rivals are. Critically, we show that this information (rivals’ respective location) can be better acquired by climbing hills. Consequently, chimpanzees do not only gather information at the moment of hearing neighbours, but our findings suggest a planning effort to gather this information.*

A second concern is that the authors generally treat the changes in the chimps' actions as if they were actively making a decision to seek out information and change their behavior according to the distribution of neighbors.  It wouldn't surprise me if chimpanzees were doing that, but it also wouldn't surprise me if they were not; perhaps they have simply learned to appreciate being high up because they are less likely to get caught unawares, without any understanding of why that is the case?  It need not have anything to do with higher order cognition.  The authors do mention this at the end of the Discussion, but rather dismissively "Although we make no claims here about social cognition used by the chimpanzees… (line 494)", especially since the entire time the chimps' actions have been referred to as "tactical," which is explicitly suggesting higher order cognition.  This possibility needs to be addressed more directly and with more acknowledgement of the fact that we

could be seeing this pattern of behavior without any tactics involved.

*In several instances, we are now discussing more deeply what could be alternatives, such as rivals’ detection form high hills as a by-product of non-territorial behaviour (L361-370), and how to tackle potential intentional territorial topography usage. See also changes L426-433, on the cognition section, talking about potential intentionality of behavior.*

In general, there are a lot of assumptions that seem as if the authors decided the chimpanzees were behaving tactically and set out to prove it, rather than seeking what the animals did.  For instance, in the abstract, the authors say that "A problem for this assertion [that tactical warfare is a driver of human evolution] is that tests in other species are absent."  Why is this a problem?  It could have emerged after the hominid lineage split from other apes and driven rapid cognitive evolution.  Again, I happen to agree with the authors that there is likely evidence in other species, but it is not a given that this statement is correct.  Then in lines 83-5 the authors introduce the hypothesis as if it were a fact and only clarify in the next paragraph that it is their hypothesis.

*We have modified those statements (abstract: L15 “Whether other animals use such tactics is unknown.”) to make them more open and less prone to self-conviction (L88-90 “Whether chimpanzees buffer the costs of territorial disputes through tactical use of high elevation to pre-emptively gather information about rivals, and use this information to make tactical decisions to approach or retreat from rivals,* *has so far not been addressed.”)*

Finally, a smaller point; if elevation is already used routinely for predator detection and signaling, why would the use of elevation in an in-group context suggest tactical usage when these other contexts do not (lines 70-2, 462-474)?  Logically, if one suggests higher order cognition, wouldn't they all?  This needs to be further addressed in the manuscript.

*We have expanded on that aspect (L79-81) by saying that “**In contrast to signaling and anti-predation, hostile rivals pose a higher cognitive challenge, particularly in fission-fusion societies in which groups coordinate their territorial activity and their numbers and movements regularly change.”*

I have a few general points on the writing as well.  The authors tend to write with lots of clauses set off with commas that can make it extremely difficult to follow the main line of argument (the first paragraph is a great example of this).  I also really like the idea of Table 1, but it needs additional text to help explain the predictions.  A reader should be able to understand it without having to read the rest of the text.  Lastly, there is a lot of repetition, particularly in the Discussion, which is quite a bit lengthier than it needs to be.  The paper in general would be more impactful if it were more concise.

*We have broken long sentences in several instances throughout the manuscript. We have now replaced that table by a graphical representation of the expectations on the tested 3-way interactions between imbalance of power, inter-community distance and elevation on the likelihood to advance towards rivals (Fig 1). Predictions regarding the stop model are embedded into the text (L97-100).*

__________________________________________________

In compliance with data protection regulations, you may request that we remove your personal registration details at any time.  (Use the following URL: <https://www.editorialmanager.com/pbiology/login.asp?a=r>). Please contact the publication office if you have any questions.
