# Supplementary material for: Chimpanzees make tactical use of high elevation in territorial contexts
Source: PLoS Biol. 2023 Nov 2;21(11):e3002350. doi: 10.1371/journal.pbio.3002350 (PMC10621857; doi:10.1371/journal.pbio.3002350)
Supplement: Attachment — Submitted filename: response to reviewers Sep2023.docx [file pbio.3002350.s022.docx]

Dear editors and reviewers,

Please find below the latest reviewers and editors’ comments, with our responses in *italic* below each comment. Line numbers refer to the submitted manuscript with track changes.

a) Please flip your Title around to include an active verb. We suggest something like "Chimpanzees make tactical use of high elevation in territorial contexts."

*We have followed this suggestion by changing the title into: “Chimpanzees make tactical use of high elevation in territorial contexts.” L1*

b) Reviewer #4 asks you to shorten the paper somewhat, to reduce repetition. While our journal does not have length restrictions, we worry that the length and repetition might compromise the appeal and accessibility of the paper, and we urge you to attend to this issue (as well as the other concerns raised by this reviewer).

*We have reduced the paper in several instances (as in each result section, by removing interpretative statements L201-204, L245-246 and L282-284), and by reducing some parts in the discussion L321, L327, L334-338, L372-373, L381-382, L398-400, L407-409, L420-422, L438-441, L448-451, L455-457, L465-469, L491-494, L500, L504-507 (mostly on the cognition and human evolution implication parts). We hope that these changes are making the paper easier to read, especially for a general audience.*

Reviewers' comments

Reviewer #4:

This is a nice revision and the authors have done an excellent job of taking into account the reviewers' earlier comments and concerns.  The manuscript is far easier to follow, which also makes it a stronger paper.  I have just a few general comments and the rest are editorial points that will help to further clarify the text.  I think that this paper is a very nice addition to the literature.

The paper is still quite long and repetitive, so if there are places in which the authors can reduce the text it would be helpful.  In particular, there is a lot of overlap between the Results and Discussion.  On the one hand, there is a lot going on in this paper, and the additional information is very useful, but if it is possible to reduce repetition, I would do so.  Second, I really like the new sections on cognition and implications for human evolution, but I would try to reduce them a bit as they are an interesting implication, rather than the main point. That being said, if the rest of the Discussion were abbreviated I might feel differently!  I don't have any specific suggestions on what to cut because I think that at this stage any decisions should be up to the authors and Editor.

*We would like to thank the editors and reviewers for their productive comments in earlier versions and for the current comments. We have tackled the issue of length by removing repetitions in several instances (as in each result section, by removing interpretative statements L201-204, L245-246 and L282-284), and by reducing some parts in the discussion L321, L327, L334-338, L372-373, L381-382, L398-400, L407-409, L420-422, L438-441, L448-451, L455-457, L465-469, L491-494, L500, L504-507 (mostly on the cognition and human evolution implication parts). We hope that these changes are making the paper easier to read, especially for a general audience.*

Lines 20-23 - this sentence is still very dense and doesn't make a whole lot of sense to someone who hasn't read the paper.  Given that this is a general interest journal and I certainly hope this paper is read by those outside of the field (it should be), please try to simplify the sentences.

*We have made the following change: L22-24 ”* *Upon leaving hills, movement decisions conformed with lowest risk engagement, indicating that higher elevation facilitates detection of rivals presence or absence.” The message is therefore more concise and clearer for a general audience.*

Line 59 - no comma after power

*The comma has been removed (L60).*

Line 64 - decision is the wrong word (decision-making?)

*Decision-making is now used instead of just decision (L65).*

Lines 67-72 - I would combine this paragraph with the above.  This paragraph is a little short to stand alone.

*We have now combined both paragraphs (starting L67-68).*

Line 94 - you don't need to say high elevation twice

*One of these statements has been removed (L95).*

Line 194 - less should be fewer (as hills are discrete objects)

*We have changed that sentence into “The number of hills previously visited had no significant effect on stop events” (L195-196). We have connected this sentence to the next one, and changed the structure of the next sentence to make it easier to understand (L197-199).*

Line 202 - neither should be nor

*Neither has been replaced by nor (L205).*

Lines 320-1 - I think you mean that they were equally likely to advance from low elevations (not equally well assessed)?

*Given the confusion of this statement, we have now replaced by “high elevation provides a tactical advantage in assessing inter-community distances, but not rival numbers”(L324). We hope it makes this sentence more explicit.*

Line 355 - the sentence starting with "given that" doesn't seem to be complete.

*This sentence is now turned into a statement on the longer of border patrols. The next sentence adds information about coordination of movements during territorial behavior, whilst the conclusive sentence links both energy expense minimization and competitive advantage of numbers. (L359-364)*

Line 477 - decision-making

*This sentence has been removed (L491).*
